# Phosphorylated Smad2 in Advanced Stage Gastric Carcinoma

**DOI:** 10.1186/1471-2407-10-652

**Published:** 2010-11-26

**Authors:** Osamu Shinto, Masakazu Yashiro, Takahiro Toyokawa, Takafumi Nishii, Ryoji Kaizaki, Taro Matsuzaki, Satoru Noda, Naoshi Kubo, Hiroaki Tanaka, Yosuke Doi, Masaichi Ohira, Kazuya Muguruma, Tetsuji Sawada, Kosei Hirakawa

**Affiliations:** 1 Department of Surgical Oncology, Osaka City University Graduate School of Medicine, 1-4-3 Asahi-machi, Abeno-ku, Osaka, Japan; 2 Oncology Institute of Geriatrics and Medical Science, Osaka City University Graduate School of Medicine, 1-4-3 Asahi-machi, Abeno-ku, Osaka, Japan

## Abstract

**Background:**

Transforming growth factor β (TGFβ) receptor signaling is closely associated with the invasion ability of gastric cancer cells. Although Smad signal is a critical integrator of TGFβ receptor signaling transduction systems, not much is known about the role of Smad2 expression in gastric carcinoma. The aim of the current study is to clarify the role of phosphorylated Smad2 (p-Smad2) in gastric adenocarcinomas at advanced stages.

**Methods:**

Immunohistochemical staining with anti-p-Smad2 was performed on paraffin-embedded specimens from 135 patients with advanced gastric adenocarcinomas. We also evaluated the relationship between the expression levels of p-Smad2 and clinicopathologic characteristics of patients with gastric adenocarcinomas.

**Results:**

The p-Smad2 expression level was high in 63 (47%) of 135 gastric carcinomas. The p-Smad2 expression level was significantly higher in diffuse type carcinoma (p = 0.007), tumours with peritoneal metastasis (p = 0.017), and tumours with lymph node metastasis (p = 0.047). The prognosis for p-Smad2-high patients was significantly (p = 0.035, log-rank) poorer than that of p-Smad2-low patients, while a multivariate analysis revealed that p-Smad2 expression was not an independence prognostic factor.

**Conclusion:**

The expression of p-Smad2 is associated with malignant phenotype and poor prognosis in patients with advanced gastric carcinoma.

## Background

Transforming growth factor β (TGFβ) is a multifunctional cytokine and one of most important pathways for cancer cells [1,2]. TGFβ binds to two different serine/threonine kinase receptors (TβR), termed type I and type II. The activated TβR type I kinase phosphorylates Smad2 and Smad3. Phosphorylated Smad2 (p-Smad2) and p-Smad3 are oligomerized with Smad4, migrate into nucleus and regulate transcription [1,3]. In normal epithelial cells, TGFβ is a potent inhibitor of proliferation, and it has been considered a tumour suppressor. Although TGFβ acts as a tumour suppressor in early-stage tumours, during tumour progression the TGFβ antiproliferative function is lost, and in certain cases TGFβ becomes an oncogenic factor inducing cell proliferation, invasion, angiogenesis, and immune suppression [4,5]. It has been reported that TGFβ can signal not only through Smad-dependent, but also Smad-independent pathways [6]. Because of the dual aspects of TGFβ in oncogenesis, Smad signal might be a critical integrator of TGFβ receptor signaling transduction systems, although the significance of Smad expression is still controversial. Bruna et al. 2007 demonstrated that high TGFβ-Smad activity is present in aggressive, highly proliferative gliomas and confers poor prognosis in patients with glioma [7], while a lack of Smad expression appears to be correlated with tumour development and poor prognosis in patients with esophageal squamous cell carcinoma [8] breast cancer [9] and colorectal cancer [10]. Not much is known regarding the prognostic value of Smad2 expression in gastric carcinoma, while several reports of serum levels of TGFβ [11,12] suggested that TGFβ can induce invasion and metastasis in gastric carcinoma. Understanding the significance of Smad2 might be useful in gastric cancer. In this study, therefore, we investigated the p-Smad2 expression of gastric carcinoma to clarify the role of p-Smad2 in advanced gastric adenocarcinomas.

## Methods

### Patients

We examined surgical samples from patients at the Osaka City University Hospital, Osaka, Japan. A total of 135 patients who had undergone resection of primary gastric tumours and were confirmed histologically to have advanced gastric cancer, were enrolled in this study. "Advanced cancer" indicates cancer invasion of the muscularis propria or serosa. None of the patients had undergone preoperative radiation or chemotherapy. Pathological diagnoses and classifications followed the Japanese Classification of Gastric Carcinoma [13]. Hepatic metastasis and peritoneal metastasis were examined at laparotomy. Peritoneal lavage cytology of the abdominal cavity was performed at laparotomy, and exfoliated cancer cells were microscopically examined. Depth of tumour invasion, differentiation, lymph node metastasis, venous invasion and lymphatic invasion were based on microscopic examination of materials obtained by surgical resection. The histological classification was based on the predominant pattern of tumour. The histological subtypes were: papillary adenocarcinoma, well-differentiated tubular adenocarcinoma and moderately-differentiated tubular adenocarcinoma, regarded as intestinal-type. The subtypes were: solid poorly-differentiated adenocarcinoma, non-solid poorly-differentiated adenocarcinoma, signet-ring cell carcinoma and mucinous carcinoma, regarded as diffuse-type. Lymph node metastasis was decided on the regional lymph nodes metastasis. The regional lymph nodes of the stomach are classified into three stations numbered as described in the Japanese classification [13] that depending upon the location of the primary tumour. As the number of increase, it indicated that spread to distant lymph nodes. The study protocol conformed to the ethical guidelines of the Declaration of Helsinki (1975). This study was approved by the Osaka City University ethics committee. Informed consent was obtained from all patients prior to entry.

### Immunohistochemical techniques

All the H&E-stained slides of the surgical specimens were reviewed, and the representative section of the tumour that included the site of deepest invasion was selected for the immunohistochemical study.

A rabbit polyclonal anti-human P-Smad2 antibody (Chemicon International, Themecula, CA. 1:2000) was used to detect p-Smad2. The methods for immunohistochemical staining of p-Smad2 have been described in the manufacturer's instructions. In brief, the slides were deparaffinized, and were heated for 20 min at 105°C by autoclave in Target Retrieval Solution (Dako, Carpinteria, CA). Sections were then incubated with 3% hydrogen peroxide to block endogenous peroxidase activity. The specimens were incubated with p-Smad2 antibody (1:2,000) overnight at 4°C. The sections were incubated with biotinylated goat anti-rabbit immunoglobulin G for 30 min, followed by three washes with PBS. The slides were treated with streptavidin-peroxidase reagent, and were incubated in PBS diaminobenzidine and 1% hydrogen peroxide v/v, followed by counterstaining with Mayer's hematoxylin. We previously reported the expression level of p-Smad2 in gastric cancer cell lines [14]. The omission of the primary antibody served as negative controls.

### Immunohistochemical determination of p-Smad2

P-Smad2 staining was evaluated at the invasion front of gastric cancers. Both hematoxylin and eosin staining were used as a reference slide to select cancer areas at the invading front. The existence of cancer cells was continuously examined from the serosa to the mucosa under a microscope. The invading front was determined at the lesion where cancer cells were first found from serosal side. P-Smad2 staining was evident in the nuclei of cancer cells. The percentage of p-Smad2 immunopositive cells was determined by counting 3 areas randomly chosen in the tissue in a total of 300 nuclei of cancer cells. Evaluation was made by two double-blinded independent observers who were unaware of clinical data and histologic diagnoses. The percentage of nucleus stained cells for p-Smad2 was also determined. There were 15 (11%) cases of a discrepant evaluation between the two independent observers. In those cases, we rechecked and discussed and the final score was obtained by consensus.

Staining was scored using the Allred scoring system as described previously [15]. For semi-quantitative analysis of p-Smad2 immuno-reactivity, the intensity of staining and percentage of tumour stained cells were evaluated in representative high-power (×400) and low-power fields (×200) using optical microscopy: intensity was scored 0-3 (0 = no immunoreactivity, 1 = weak, 2 = moderate, and 3 = intense immunoreactivity) and proportion was scored 0-5 (0 = 0%; 1 = 1-20%; 2 = 21-40%; 3 = 41-60%; 4 = 61-80%; 5 = 81-100%). The two scores were then added to obtain the final result of 0-8. The median score of all cases was 6.07. Then the cut-off point was determined 6; p-Smad2 expression was considered high expression when scores were ≥7 and low expression when scores were <7.

### Statistical analysis

Comparative analyses of the data were performed using the Chi-square test or Fisher's exact test. The survival durations were calculated using the Kaplan-Meier method and analyzed by the log-rank test to compare the cumulative survival durations in the patient groups. Cox proportional-hazards regression was used to compute univariate and multivariate hazards ratios for the study parameters. A p-value of less than 0.05 was defined as being statistically significant. SPSS 10.0 software (SPSS Japan, Tokyo, Japan) was used for the analyses.

## Results

### Relationship between clinicopathological features and phospho-Smad2 expression

**Figure 1:**
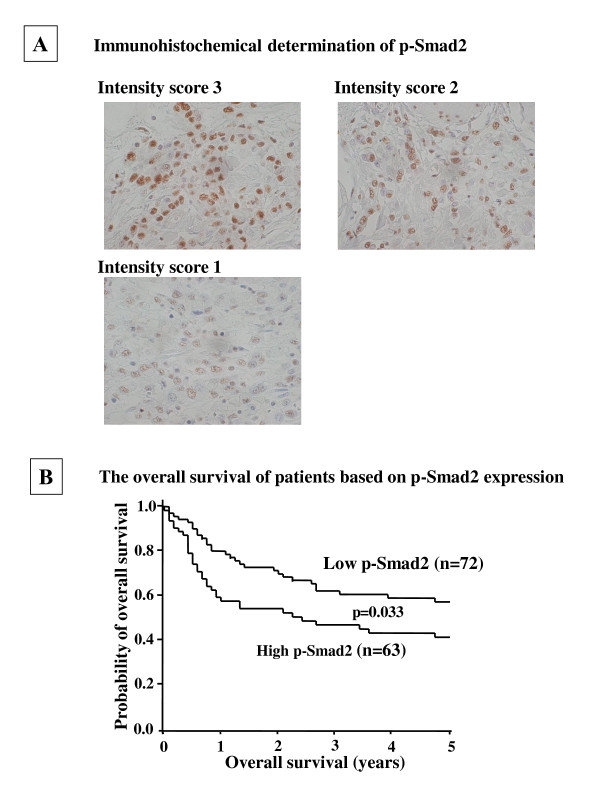
**(A), Immunohistochemical determination of p-Smad2**. P-Smad2 was found in the nuclei of cancer cells. The Immunoreactive intensity score of p-Smad2 staining in tumour cells was evaluated using the positive and negative controls. Score 3 means high intensity as same as the positive control. Immunoreactivity, score 1 = weak, score 2 = moderate, and score 3 = intense. (×400). (**B**), The overall survival of patients based on p-Smad2 expression. The Kaplan-Meier survival curve shows the overall survival in relation to the p-Smad2 expression in 135 patients with gastric carcinoma. A statistically significant difference in the survival was observed between the high p-Smad2 groups and low p-Smad2 groups (p = 0.035; log-rank,).

Phospho-Smad2 was mainly immunolocalized in the nuclei of cancer cells (Figure 1A). The intensity of staining and percentage of stained tumour cells were variable. Regarding intensity, score 0 was found in no cases (0%), score 1 in 55 cases (41%), score 2 in 33 cases (24%), and score 3 in 47 (35%) cases. Upon evaluation of positive cell percentages, score 0 was found in no cases (0%), score 1 in 2 cases (1%), score 2 in 11 cases (8%), score 3 in 16 cases (12%), score 4 in 43 cases (32%), and score 5 in 63 cases (47%). The total Allred scores varied from 2 to 8. The high expression level of p-Smad2 was found in 63 (47%) of 135 gastric carcinomas. The relationships between p-Smad2 expression and clinicopathological features of the tumours are shown in Table 1. P-Smad2 expression level was significantly high in diffuse type carcinoma (p = 0.011) and significantly correlated with peritoneal metastasis (p = 0.017), lymph node metastasis by Japanese classification [13] or UICC classification [16] (p = 0.047, p = 0.004) and peritoneal cytology (p = 0.026). "Peritoneal cytology" means peritoneal lavage cytology at laparotomy as a standard method for the detection of free tumor cells and a useful predictor of peritoneal recurrence in gastric cancer. In 12 cases, peritoneal cytology was not performed. Then the total number of peritoneal cytology was 123 cases. There was no statistically significant association between the phospho-Smad2 expression and either hepatic metastasis, venous invasion or lymphatic invasion. The number of metastatic lymph nodes in high-expression group (median = 12.3) was significantly (p = 0.002) higher than that in low-expression group (median = 5.77), while no difference of the number of affected lymph nodes was shown between low-p-Smad2 groups (median = 41.1) and high-p-Smad2 groups (median = 44.4).

**Table 1:** Relationships between p-Smad2 and clinicopathological features in 135 gastric cancer cases.

Parameter		P-Smad2		*p*-Value
			
		High (n = 63)	Low (n = 72)	
Gender				
	Male	39 (41.9%)	54 (58.1%)	N.S.
	Female	24 (57.1%)	18 (42.9%)	
Morphologic feature^+^				
	Type 1	6 (37.5%)	10 (62.5%)	0.025
	Type 2	14 (35.0%)	26 (65.0%)	
	Type 3	17 (45.9%)	20 (54.1%)	
	Type 4	26 (61.9%)	16 (38.1%)	
T stage				
	2	7 (25.9%)	20 (74.1%)	0.031
	3	52 (50.0%)	52 (50.0%)	
	4	4 (100.0%)	0 (0.00%)	
Differentiation				
	Intestinal-type	15 (31.2%)	33 (68.8%)	0.011
	Diffuse-type	48 (55.2%)	39 (44.8%)	
Lymph node metastasis^+^ (Japanese classification)	N0 and N1	34 (40.5%)	50 (59.5%)	0.047
	N2 and N3	29 (56.9%)	22 (43.1%)	
Lymph node metastasis^++^ (UICC classification)	N0 and N1	31 (36.9%)	53 (63.1%)	0.004
	N2 and N3	32 (62.7%)	19 (37.3%)	
Hepatic metastasis				
	Negative	62 (47.0%)	70 (53.0%)	N.S.
	Positive	1 (33.3%)	2 (66.7%)	
Peritoneal metastasis				
	Negative	48 (42.1%)	66 (57.9%)	0.017
	Positive	15 (71.4%)	6 (28.6%)	
Peritoneal cytology^+++^				
	Negative	36 (40.4%)	53 (59.6%)	0.026
	Positive	22 (64.7%)	12 (35.3%)	
Lymphatic invasion				
	Ly0 and ly1	26 (40.6%)	38 (59.4%)	N.S.
	Ly2 and ly3	37 (52.1%)	34 (47.9%)	
Venous invasion				
	Negative	50 (45.5%)	60 (54.5%)	N.S.
	Positive	13 (52.0%)	12 (48.0%)	
Clinical stage				
	I	2 (22.2%)	7 (77.8%)	0.022
	II	15 (39.5%)	23 (60.5%)	
	III	18 (40.0%)	27 (60.0%)	
	IV	28 (68.7%)	15 (31.3%)	

^+, ^Classification according to the General Rules for Gastric Cancer Study of the Japanese Research Society for Gastric Cancer [13]. Type 1 is defined as a polypoid tumor; type 2 as polypoid tumor with ulceration and with sharply demarcated margins; type 3 as ulcerated carcinoma with cancer infiltration into the surrounding wall; type 4 as diffusely infiltrating flat carcinoma in which ulceration is usually not a marked feature.

^++, ^Nodal stage was assessed by the Union Internationale Contre le Cancer (UICC) tumor node metastasis (TNM) classification [16] and by classification according to the general rules for gastric cancer study of the Japanese Research Society for Gastric Cancer. In the TNM classification, nodal stage was classified according to the number of involved regional LNs as follows: pN0, no LN spread; pN1, 1 to 6 diseased LNs; pN2, 7 to 15; and pN3, more than 15. In the Japanese classification, nodal stage was classified according to the extent of positive LN into n0, n1, n2, and n3 and is considered to reflect the anatomic pathway of lymphatic spread. n1 is defined as proximate regional LN disease, and n2 or n3 are defined as distant.

**^+++, ^**In 12 cases, peritoneal cytology was not performed (Total 123 cases).

### Phospho-Smad2 relates with poor prognosis in gastric cancer

In this series of advanced gastric cancer, survival of phospho-Smad2 high level expression patients was significantly poorer than that of phospho-Smad2 low level expression patients (p = 0.035, Figure 1B). In univariate analysis, high phospho-Smad2 expression (p = 0.048), morphological type 4 (p < 0.001), diffuse-type tumours (p < 0.001), lymph node metastasis (p < 0.001), peritoneal metastasis (p < 0.001), peritoneal free cancer cells (p < 0.001), lymphatic invasion (p = 0.006) and clinical stage IV (p < 0.001) were significantly associated to poor patient survival (Table 2). In multivariate analysis, morphologic type 4, diffuse-type tumours and clinical stage IV were statistically independent prognostic factors, but phospho-Smad2 was not (Table 3).

**Table 2:** Univariate analysis with respect to overall survival (n = 135).

Parameter	Risk ratio	95% Confidence interval	*p*-Value
P-Smad2 expression	1.650	1.005-2.710	0.048
Low *vs*. high			
Morphologic feature	5.354	3.199-8.963	< 0.001
Type 1, 2, and 3 *vs*. type 4			
Differentiation	3.883	2.020-7.463	< 0.001
Intestinal-type *vs*. Diffuse-type			
Lymph node metastasis (Japanese classification)	3.269	1.978-5.403	< 0.001
N0 and N1 *vs*. N2 and N3			
Peritoneal dissemination	5.534	3.056-8.738	< 0.001
Negative *vs*. positive			
Peritoneal cytology	5.937	3.468-10.166	< 0.001
Negative *vs*. positive			
Lymphatic invasion	2.055	1.234-3.421	0.006
Ly0 and ly1 *vs*. ly2 and ly3			
Clinical Stage	6.901	4.088-11.651	< 0.001
I, II, and III *vs*. IV			

**Table 3:** Multivariate analysis with respect to overall survival (n = 135).

Parameter	Risk ratio	95% Confidence interval	*p*-Value
P-Smad2 expression	0.627	0.347-1.133	0.122
Low *vs*. high			
Morphologic feature	2.325	1.221-4.388	0.010
Type 1, 2, and 3 *vs*. type 4			
Differentiation	2.807	1.251-6.298	0.012
Intestinal-type *vs*. Diffuse-type			
Lymph node metastasis (Japanese classification)	1.387	0.689-2.794	0.360
N0 and N1 *vs*. N2 and N3			
Peritoneal dissemination	1.097	0.549-2.193	0.793
Negative *vs*. positive			
Lymphatic invasion	1.394	0.805-2.413	0.235
Ly0 and ly1 *vs*. ly2 and ly3			
Clinical Stage	4.382	1.924-9.978	< 0.001
I, II, and III *vs*. IV			

## Discussion

P-Smads2 is a primary step and intracellular signaling effector for the mediation of intracellular signaling of TGFβ [1]. In early-stage tumours TGFβ acts as a tumours suppressor, while in advanced tumours it becomes an oncogenic factor. Because of the dual role of TGFβ in oncogenesis, depending on the type and stage of the tumour, we focused our study on p-Smad2 expression on the advanced stage of gastric cancer. P-Smad2 expression levels of cancer cells were high in 63 (47%) of 135 gastric carcinomas, suggesting that some types of gastric carcinomas were strongly-affected by TGFβ. In the present study, p-Smad2 expression in tumour cells was significantly higher in diffuse-type tumours and in cases with peritoneal metastasis, free peritoneal cancer cells and lymph node metastasis. Moreover, p-Smad2 high expression predicted poor survival. These findings suggest that p-Smad2 is associated to malignant phenotype and is a molecular biomarker of disease outcome in advanced gastric cancer.

P-Smad2 expression levels of cancer cells were high in diffuse-type gastric carcinoma also known as a scirrhous type. There are two types of gastric cancer: diffuse type and intestinal type, according to the Laurén classification [17]. The characteristic clinical features of diffuse-type gastric carcinoma, a diffusely infiltrating type of gastric carcinoma, include a high frequency of metastasis to the LNs [18-20] and to the peritoneum [21-23]. The expression level of TGFβ in scirrhous gastric carcinoma was related with poor prognosis [24]. Tumor cells in scirrhous carcinoma produce more TGFβ than non-scirrhous carcinoma [25,26]. TGFβ signals might play an important role for the metastatic spread of cancer cells such as migration, and invasion, as previously reported [2,11,27]. In fact, Overexpression of Smad2 was associated with metastasis, and was correlated with poor prognosis of gastric tumors especially diffuse-type gastric carcinoma. TGFβ-Smad2 signaling may have an important role in the progression of diffuse-type gastric carcinoma. The activated Smad2 level might be associated with the different clinical phenotype of malignancy between the diffuse-type and intestinal-type in gastric carcinoma.

Immunohistochemical is a good method of choice for the evaluation of protein expression within a particular tissue, since it is possible to distinguish the cell types stained, as well as the number of cells and intensity of the immunohistochemical stained. Both analyses must be combined and it was done. Allred score system evaluates a combination of intensity and proportion, potentially increasing the prognostic value of the analysis by the intensity alone or the proportion alone in this study. Allred scores of 7-8 were considered suitable for the identification of aggressive advanced gastric carcinoma. Allred score system for p-Smad2 expression might be suitable for accurately assessing p-Smad2-expressing tumor cells, and might be adequate for predicting the outcome of patients with gastric cancer. P-Smad2 staining was evaluated at the invasion front of gastric cancers. P-Smad2 expression in nuclei of cancer cells was continuously examined from the serosa to the mucosa under a microscope. Few difference of p-Smad2 staining was found between infiltrative front and surface lesions in the same cases. And, the tumor cells at the invading front might play an important role for distant metastasis. Therefore, p-Smad2 expression of the tumor cells was evaluated at the invading front.

Since the activated Smad2 was associated with the malignant phenotype of gastric cancer, it is possible that inhibition of p-Smad2 signaling in gastric carcinoma may yield beneficial effects through inhibition of invasion and metastasis of cancer. Although, the TGFβ pathway is being evaluated as a therapeutic target in gastric cancer [28,29], the complex roles of TGFβ in tumor progression might make it difficult to select the patients that will benefit from an anti-TGFβ therapy. The determination of p-Smad2 expression by the Allred score system might improve patient selection and the development of successful targeted therapies, although it will be necessary to confirm that the p-Smad2 Allred score also provides significant prognostic power for gastric cancer patients in a prospective study.

## Conclusion

P-Smad2 appears to play a crucial role in advanced gastric carcinoma and may be a useful prognostic marker of poor prognosis.

## Competing interests

The authors declare that they have no competing interests.

## Authors' contributions

OS conceived the design of the study, performed the statistical analysis and drafted the manuscript. MY conceived the design of the study and performed the statistical analysis. TT, RK, TN, TM, SN, and YD performed the statistical analysis and collected the material sampling. NK, HT, MO, KM, and TS collected the material sampling. KH participated in its design and coordination.

### Sources of support

This study was supported in part by Grants-in Aid for Scientific Research (Nos. 18591475, 20591073, and 18390369) from the Ministry of Education, Science, Sports, Culture and Technology of Japan, by a JSGE Grant-in Aid for Scientific Research, by a Grant-in Aid for Kobayashi Foundation for Innovative Cancer Chemotherapy, by a Grant-in Aid for the Sagawa Foundation for Cancer Research, and by a Grant-in Aid for the Osaka Medical Research Foundation for Incurable Diseases

## Pre-publication history

The pre-publication history for this paper can be accessed here:

http://www.biomedcentral.com/1471-2407/10/652/prepub
